# Groundwater recharge potential zonation using an ensemble of machine learning and bivariate statistical models

**DOI:** 10.1038/s41598-021-85205-6

**Published:** 2021-03-10

**Authors:** Maryam Sadat Jaafarzadeh, Naser Tahmasebipour, Ali Haghizadeh, Hamid Reza Pourghasemi, Hamed Rouhani

**Affiliations:** 1 Department of Watershed Management Engineering, Faculty of Agriculture, Lorestan University, Khorramabad, Iran; 2 Department of Natural Resources and Environmental Engineering, College of Agriculture, Shiraz University, Shiraz, Iran; 3 Department of Range and Watershed, Management, College of Agriculture Science and Natural Resource, Gonbad-e-Kavous University, Golestan, Iran

**Keywords:** Environmental sciences, Hydrology

## Abstract

Many regions in Iran are currently experience water crisis, largely driven by frequent droughts and expanding agricultural land combined with over abstraction of groundwater. Therefore, it is extremely important to identify potential groundwater recharge (GWR) zones to help in prevent water scarcity. The key objective of this research is to applying different scenarios for GWR potential mapping by means of a classifier ensemble approach, namely a combination of Maximum Entropy (ME) and Frequency Ratio (FR) models in a semi-arid mountainous, Marboreh Watershed of Iran. To consider the ensemble effect of these models, 15 input layers were generated and used in two models and then the models were combined in seven scenarios. According to marginal response curves (MRCs) and the Jackknife technique, quaternary formations (Qft1 and Qft2) of lithology, sandy-clay-loam (Sa. Cl. L) class of soil, 0–4% class of slope, and agriculture & rangeland classes of land use, offered the highest percolation potential. Results of the FR model showed that the highest weight belonged to Qft1 rocks and Sa. Cl. L textures. Seven scenarios were used for GWR potential maps by different ensembles based on basic mathematical operations. Correctly Classified Instances (CCI), and the AUC indices were applied to validate model predictions. The validation indices showed that scenarios 5 had the best performance. The combination of models by different ensemble scenarios enhances the efficiency of these models. This study serves as a basis for future investigations and provides useful information for prediction of sites with groundwater recharge potential through combination of state-of-the-art statistical and machine learning models. The proposed ensemble model reduced the machine learning and statistical models’ limitations gaps and promoted the accuracy of the model where combining, especially for data-scarce areas. The results of present study can be used for the GWR potential mapping, land use planning, and groundwater development plans.

## Introduction

Over the past half century, the least developed countries population has grown rapidly, and continues to grow slowly over the coming decades^1^. This can lead to a further increase in agricultural activities and consequently, putting unprecedented pressure on the surface water and groundwater resources. Over-extraction of groundwater may result in falling groundwater level^2^, land-subsidence^2,3^, salinization^4^, reduced well-yields^5^, increased pumping costs^6,7^, and enhances saltwater intrusion^7^. The result of all these changes was the reduction the surface of 60% of major aquifers in many parts of the world^8^; additionally, one of the great challenges in the twenty-first century is the change in the global weather patterns which likely impose additional pressures^9,10^. Another problem concerned increase in water demand, which dropped access to adequate water to meet public needs^11,12^.

Groundwater is one of the sources of water stock which can be used to tackle the problem of water scarcity^13,14^. The social value of groundwater should not be measured by the volume of withdrawal^12^. Due to its availability on a local scale, the possibility of adjusting withdrawal in response to demand fluctuations, high reliability in periods of drought, and desirable quality with minimal need for purification, groundwater has higher economic value compared to surface water. Therefore, identification of groundwater recharge zones is a critical for the sustainable management of groundwater resources^15–19^. Groundwater recharge is commonly determined by the amount of precipitation and the percolation process. Recharge occurs when water moves through the unsaturated zone^20^. In semi-arid areas with dry and wet seasons, recharge occurred occasionally after high rainfall. Therefore, problems caused by droughts on the one hand, and devastating floods on the other, highlight the need for proper water resource management. In this regard, collecting surface water and identifying the groundwater recharge zones for storing water are the most important strategies for water resource management, particularly in semi-arid areas of Iran.

There are several approaches to groundwater characteristics and groundwater recharge assessment. With the variety of techniques available for recharge assessment, selecting the appropriate technique is often not easy. Implementing some of these techniques may be restricted by some factors such as the resources available, precision level needed^21^, the space and time scales, reliability/range of recharge evaluation, as well as time and cost^22,23^. The methods can generally be categorized as being either direct or indirect. Direct methods include geological and geophysical explorations (seismic, sonic and magnetic) and drilling tests^21^. These methods in-situ investigation is costly and not feasible for estimation at watershed scale. Indirect methods comprise physical and numerical modeling, physically based spatially distributed method^21^ and tracer. Most direct approaches provide information over relatively small scale^24^ and the methods often consider on a single affecting factor for GWR^25^. Tracer methods for Groundwater recharge assessment are considered the most promising approach but these methods are generally expensive, time-consuming, and often need to be integrated due to the large size of data^23^. Numerical models simplify the physical process of recharge and requires a certain measure parameter to obtain reliable prediction but the model accuracy relies on possible error in initial data, computational costs and some parameters are rarely available in developing countries. Moreover, these models involved many parameters that makes it hard to obtain a unique solution^26^.

Numerous geospatial techniques have been widely used in the past decade and continue to develop new techniques especially due to their capability to increase spatial and temporal information. A series of studies have been conducted by researchers based on knowledge-driven modeling approach or multi influencing factor with integrating various thematic maps on the GWR potential zones using GIS^24,27–32^. Rajaskhar et al.^7^ and Dar et al.^33^ used AHP (Analytic Hierarchy Process) for artificial recharge sites identification, as well as GWR potential zones in Anantapour and Kashmir Valley, respectively in India. The weights of several thematic maps include geomorphology, slope, drainage density, land use, lithology, lineament density, rainfall, and soil texture were calculated by the AHP. This method utilizes expert-based opinion and has relatively high potential for error^34^. For example, Mogaji et al^35^ compared AHP and Evidential Belief Function (EBF) for identification of GWRP zones in southwestern Nigeria. They concluded that based on the area under the curve (AUC) measure, EBF, as a data driven technique, outperformed the AHP. Chenini and Msaddek^36^ compared the bivariate statistical analysis and logistic regression model (LR) to produce a GWRP map in Qued Guenniche in the north of Tunisia. The authors found that the bivariate and multivariate statistical approaches provide more accurate results than the AHP. On the other hand, data-driven modeling which is based on statistical and machine learning algorithms can be identified and mapped effectively behavior of phenomena. Therefore, recently, there has been a trend towards using numerous statistical methods and machine learning methods to map the GWR potential. Moving towards more advance algorithms in GWR potential mapping, Pourghasemi et al^37^ used machine learning algorithms, namely support vector machines (SVM), multivariate adaptive regression splines (MARS), and random forest (RF) to map groundwater recharge potential zones in Firuzkuh County, Iran. In this research, infiltration rate was measured using double ring infiltrometer with random permeability sampling at eleven lithological types. In an attempt to develop sophisticated approaches in phenomena mapping, researchers have developed ensemble models with combining statistical models and machine learnings techniques in spatial prediction of natural phenomena which improve prediction accuracy. In other words, higher precision and predictive ability in comparison with individual machine learning models is the significant property of ensemble models^38,39^. To overwhelming literatures on susceptibility and potential mapping of natural phenomena studies such as gully erosion^40^, groundwater^41–43^, flood^44,45^ and landslide^46–48^ are rich of researches. The advantage of the ensemble methods is that achieve higher prediction accuracy by incorporating ideas of multiple learners and reduce bias and variance.

Iran has one percent of the world’s population but the country contains only 0.63 percent of the world’s renewable fresh water. In terms of climatic conditions major parts of Iran have been considered as arid and semi-arid regions. In addition to low and spatial and temporal distribution of precipitation, the occurrence of precipitation with rather high severity, which cause heavy rainfall and destructive floods is another important feature of these regions. Declining groundwater level in arid ad semi-arid area like Iran, where groundwater provides approximately 80% of the water supply for agricultural and households^49^ become a serious threat to sustainability. In these areas, extra exploitation of aquifers in comparison with natural recharges has caused depletion in major aquifers. The semi-arid terrains of Iran such as Varamin, Parishan, Qazvin, Darab, plains are among the most constantly increasing agricultural cultivated land areas for maize, cotton, wheat cultivation. The stable growth in agriculture is possible by irrigation with shallow local groundwater resources^50^. However, intense groundwater abstraction with limited recharge, resulting in aquifer depletion and increasing pumping costs^51^. Moreover, climate change is expected to impose additional limits to water supply^9^. Over the last half century, the groundwater withdrawals in Iran has tripled. According to SZCEC the groundwater levels have declined from by nearly 1 m in the south western to 3 m in the northern and northeastern parts of the Marboreh watershed for the last 20 years (SZCEC, 2012) and negative social and environmental effects becoming increasingly evident.

Due to the importance of recharge zones, the identification of appropriate recharge zone is critical for managing aquifer recharge. So far, the ensemble method is applied in different spatial phenomena mapping but to our knowledge no prior studies have been applied in GWR potential zones mapping. Therefore, the present study aimed to evaluate prediction performance ensemble models based on FR and Maximum Entropy (ME) to identify GWR potential mapping in a mountainous watershed, Marboreh in Lorestan Province, Iran, where the groundwater dependency has been largely increased over the last decades. Specifically, the machine learning (ME) and a bivariate statistical method (FR) were used for assess ensemble of spatial prediction of GWR potential scenarios. Whereas, Jackknife test as a simple but effective method utilized for screening important casual factors based on the random sampling in training dataset. The performance of spatial prediction models was compared by area under curve (AUC) and Correctly Classified Instances (CCI).

## Methodology

### Study area

**Figure 1:**
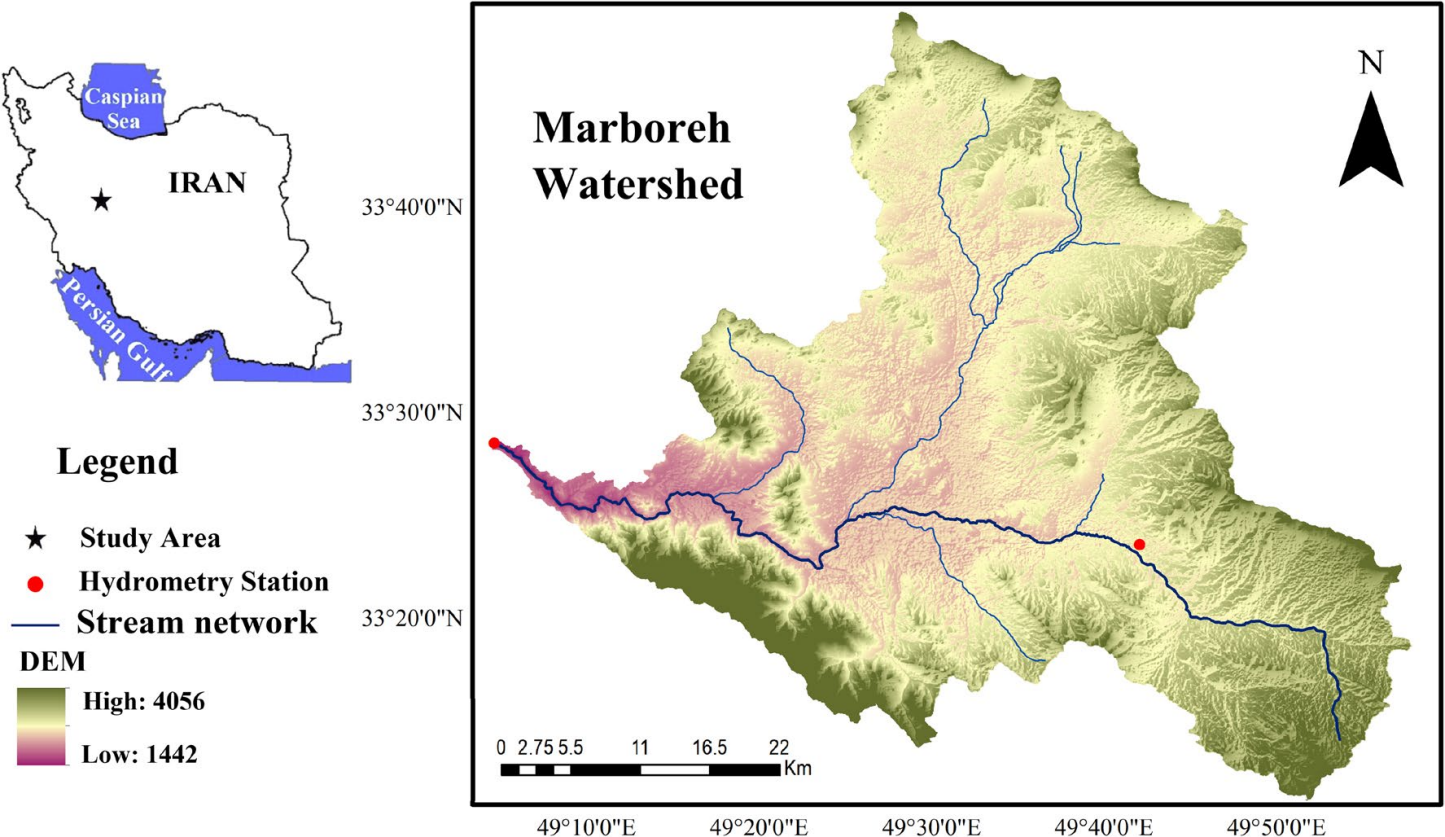
Location of the study area in Lorestan Province, Iran.

Marboreh Watershed is located between 33^o^12′ to 33^o^51′ N and 49^o^03′ to 49^o^57′ E in the west of Iran, Lorestan Province (Fig. 1). The total area of Marboreh Watershed is about 2560 km^2^ with a cold semi-arid climate. The annual rainfall decreases from south (900 mm) towards eastern (280 mm) parts of the watershed with the average annual rainfall and the average annual temperature of 412 mm and 13.8 ℃, respectively (LPMO (Lorestan Province Meteorological Organization.)). Rainfall is not uniformly distributed through the year, with the majority occurring in March and April. In the summer time, the rainfall mostly occurs once or twice nearly every month. Topographically, the region is a part of Zagros Chain with altitude range from 1442 to 4056 m, with average altitude of 2749 m. The main river, Marboreh, originates in the Aligudarz Mountains at the east of watershed, making its 110.22 km journey westwards along the entire length study area. Smaller tributaries contributing to the river discharge such as Azna, Aligudarz, and Kamandan. Finally, Marboreh River joins to Tireh River and form the Sezar, which is one of the main branches of Dez basin that flows in Karun River, the longest river in Iran which finally drains into the Persian Gulf. Geologically, the watershed lies within Zagros Fold and 29 types of geological formation cover this watershed, and the dominate land use is agriculture (49.5%), followed by rangeland (42%).

**Figure 2:**
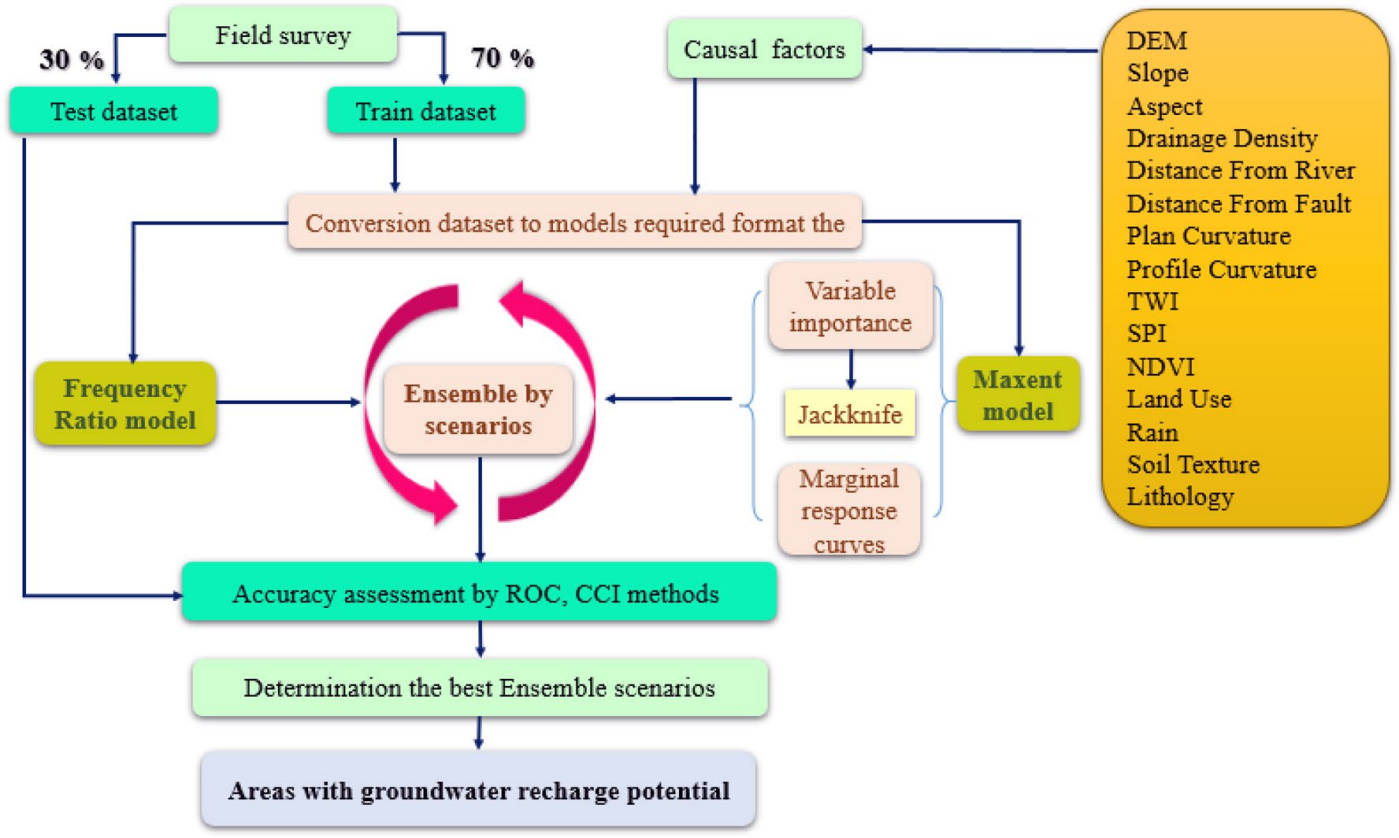
Flowchart of carrying out the methodology in this study.

The flowchart shown in Fig. 2, illustrate the method employed in this study and briefly given in the following sections:

### Permeability samples inventory map

**Figure 3:**
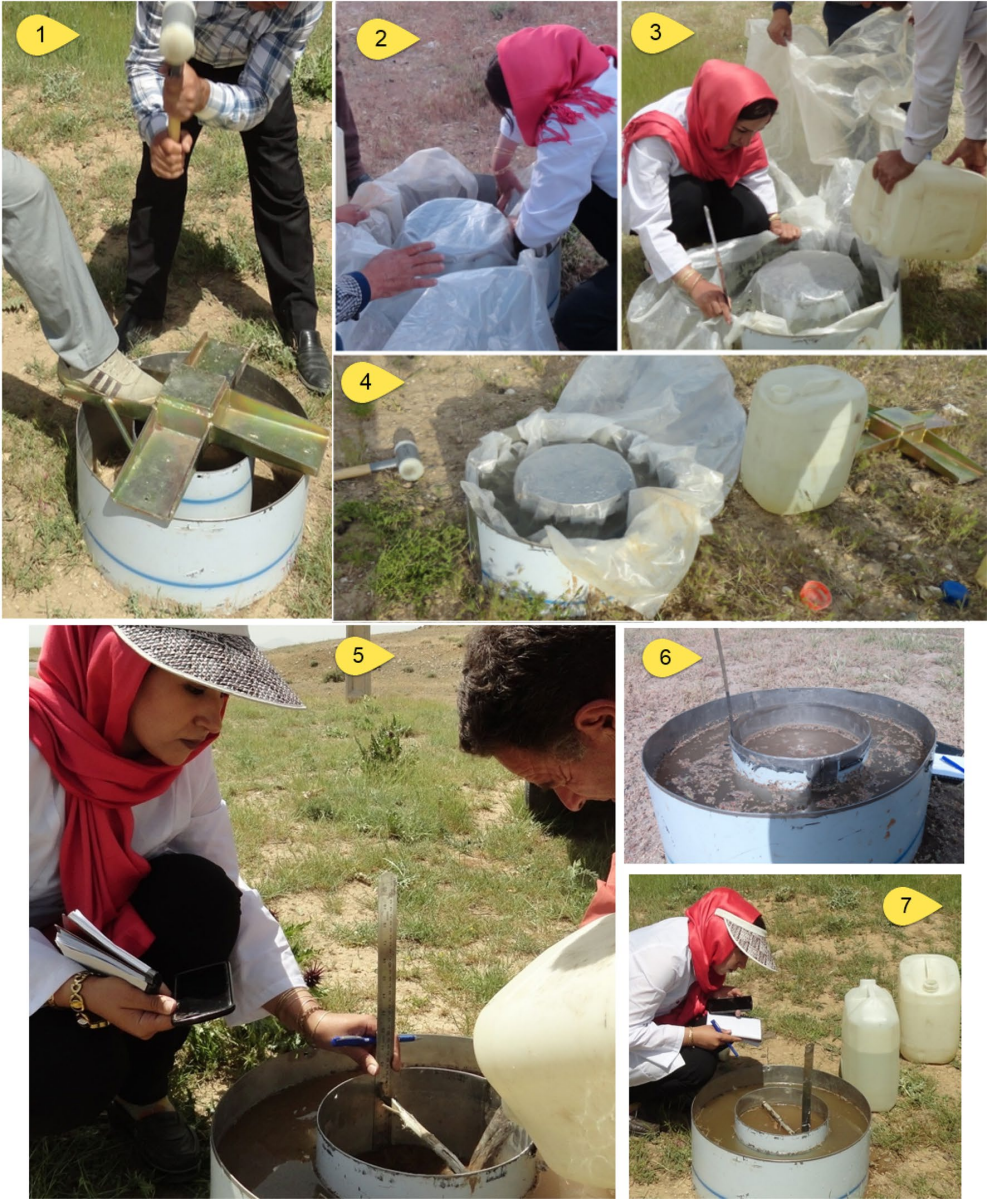
Double-ring infiltrometer method in the study area.

The key link between surface water and groundwater is groundwater recharge^4^ and quantifying the Groundwater recharge is difficult at large scale. On the other hand, the gross groundwater recharge rate may be depending on the permeability and soil’s absorption, which make it easier to measure^52^. In order to evaluate the performance of the models in GWR potential mapping, double-ring infiltrometer method and soil sampling (Fig. 3) were used for understanding the spatial variability of GWR^37,53^. The double-ring infiltrometer consist of an inner and outer ring that are driven 5-cm into the subsurface ground. The inner and the outer rings were filled with water. The head is kept constant during the experiments. The procedure continue until the infiltration rate is considered steady and set up by the Sangab Zagros Consulting Engineering Company^22^ standard. Due to the extent of the studied area and the cost of the sampling process, information from previous studies^22^ in the studied area were also used. The percolation points were randomly split into two datasets: the first group for model training (70%) and to validate the training dataset, a separate independent set of data (30%) to evaluate modelled and observed maps.

**Figure 4:**
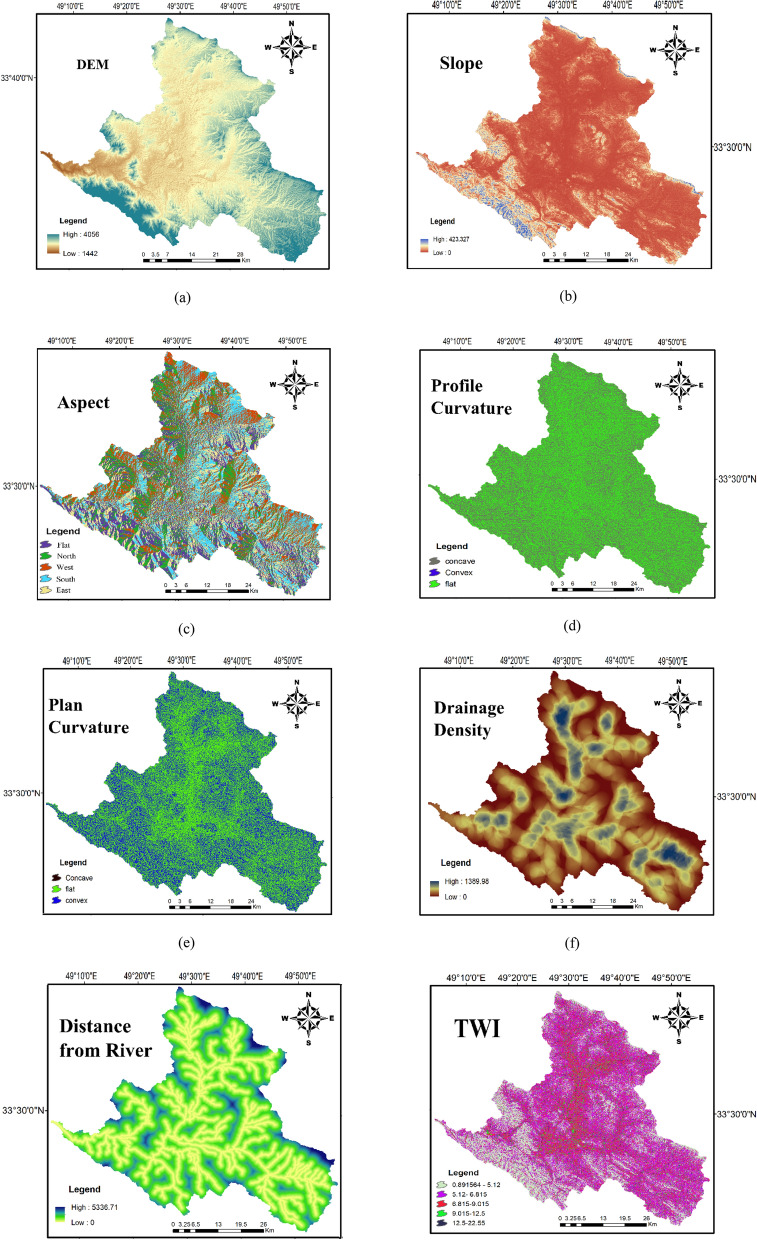

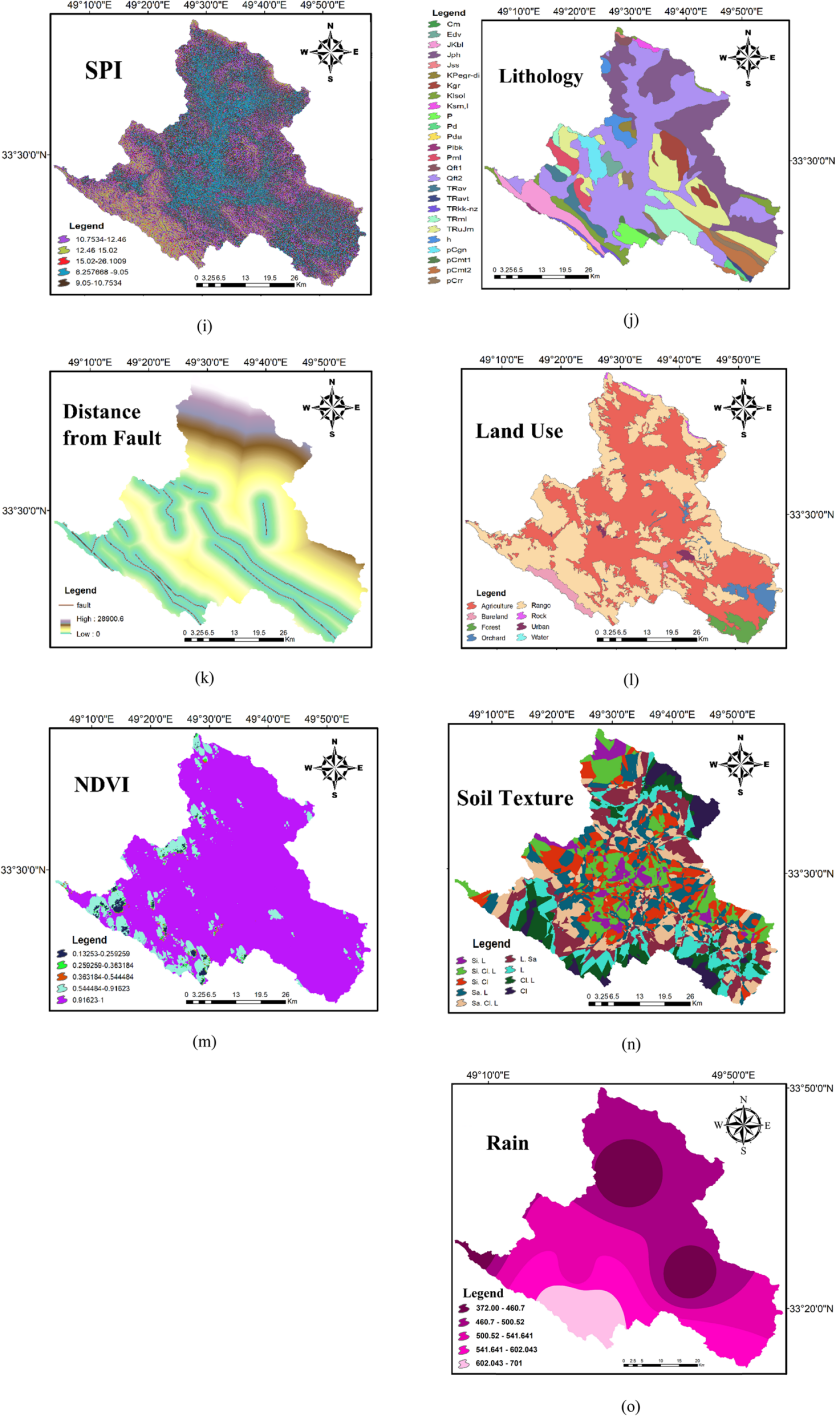
The condition factors maps used in FR and ME models (maps layouts were prepared in ArcGIS 10.6 software).

### GWR influencing factors

GWR vary significantly in different places depending on topographic factors, hydrogeology variables, and climate conditions^25,54^. Consequently, based on various studies^25,27,37,54,55^ and data availability, 15 effective factors on GWR potential, namely elevation, slope percent, slope aspect, profile curvature, plan curvature, drainage density, distance from rivers, topographic wetness index (TWI), stream power index (SPI), Rainfall, lithology, fault distance, land use (LU), Normalized Difference Vegetation Index (NDVI), and soil texture were used. Out of these selected thematic maps, elevation, slope degree, slope aspect, drainage density, TWI and SPI were generated from ASTER DEM data with a pixel size of 30 × 30-m, while the remaining maps were extracted from Landsat-8 images and through conventional data, such as soil and geology data.

#### Topographic influencing factors

Elevation play in key role on vegetation and climate that have connection with recharge distribution area. Here, the elevation ranges from 1442 to 4056 m, and the hypsometric map extracted from DEM and classified into five classes (Fig. 4a). In groundwater recharge investigations, slope angel is a major determinant factor and highlights the importance of topography and the size of runoff-saturated zones^56,57^. The regions of steep slope facilities high runoff, whereas at the lower slope gradients, runoff generation reduced and eventually increase infiltration rate and recharge the saturated zone^58^. The slope map extracted from DEM and was classified into 5 classes (Fig. 4b). Slope aspect influences GWR as indicator for solar radiation which strongly influences infiltration rate^54^. Slope aspect is the orientation of slope, measured clockwise in degrees from 0 to 360. This factor was derived from the DEM in ArcGIS10.6 (https://www.arcgis.com) and classified into five main categories (Fig. 4c). Flow distribution on the surface depends on topography, which is represented by profile curvature. Profile curvature influences on the overland flow velocity, whereas plan curvature mainly affects flow convergence and divergence (Fig. 4d–e).

#### Hydrological influencing factors

Drainage density is another effective factor for controlling GWR. The drainage density demonstrates the signature of surface to subsurface formation^59^ and it implies indirectly soil characteristics, which is assigned soil infiltration capacity^55^. Drainage density was extracted from DEM with help of ArcHydro Tool of ArcGIS10.6. Then the extracted drainage networks (km^-1^) were overlaid on vector digitized stream map of LPRWA (Lorestan Province Regional Water Authority.) for improved drainage direction map and the area was categorized into five classes (Fig. 4f). The low drainage density indicates low surface runoff and hence high infiltration rate^57^ and consequently a high GWR potential. Rivers are one of the sources of GWR, and thus influence GWR potential in a watershed. The Euclidean distance tool from spatial analyst tools in ArcGIS10.6 was used to generate distance categories. The distance map was reclassified into five classes (Fig. 4g).

Topographic wetness index (TWI) and stream power index (SPI) are as secondary topographic indices. In the GWR potential zone, one of the important secondary topographic factors was TWI. TWI was proposed by Moore et al. (1991) to indicate local groundwater potential. TWI relies on upslope area which highlights the potential exfiltration groundwater by topography effects; the lower TWI, the greater GWR potential in the specific class. The TWI of the region ranges from 0.891 to 22.553 and the TWI was further classified in five classes (Fig. 4h). SPI index describes stream power and its value ranges from 6.257 to 26.100. Five categories of SPI values were generated based on the natural break method as (< 9.04), (9.04–10.75), (10.75–12.46), (12.46–15.02), and (> 15.02). (Fig. 4i). The mean annual rainfall was used to determine the groundwater recharge investigations of the Marboreh watershed. The mean annual rainfall over the study area was interpolated based on the Kriging method from 12 gauging station. The mean annual rainfall ranges from 280 to 900 mm (Fig. 4o.).

#### Geological influencing factors

The geological factors generally demonstrate the distribution of various rock units, which have a notable effect on GWR and water availability through hydraulic conductivity^60^. In this study, the lithological map was extracted from the national geological map of Iran at a 1:100,000-scale, and encompassed 26 major lithological groups (Fig. 4j). Fault map (Fig. 4k) was extracted from the national geological map of Iran at a 1:100,000-scale. This layer was used to infer groundwater storage and in regional scale, ground water flow direction is controlled by fault systems.

#### Ecological influencing factors

Land use (LU), NDVI, and soil are ecological parameters, which are commonly used factors for GWR potential zones^27,37,54,55^. The LU types can be significantly effective on runoff, permeability, and evapotranspiration. As stated by Gee et al^61^, recharge in vegetated areas is much lower than non-vegetated areas. In addition, recharge is greater in agricultural lands and grass lands than perennial lands including, shrub and forest areas^62^. The Landsat8 data (downloaded from https://www.earthexplorerusgs.gov; column 165 and row 37, 17 June 2018) were used to determine the types of LU. Also, LU was classified by a supervised classification technique according to the maximum likelihood approach into 8 classes, namely; agriculture, bare land, forest, orchard, rangeland, rocky areas, urban, and water. LU within the study area is dominated by agriculture (49.6%) and rangeland (43.4%) (Fig. 4l). The vegetation density and coverage were represented using the Normalized Difference Vegetation Index (NDVI) map. The NDVI layer was prepared in ENVI 5.3. The value range of NDVI is -1 to 1, and higher value of NDVI represents dense vegetation. NDVI values were grouped based on the natural break method into five classes, namely (< 0.11), (0.11–0.17), (0.17–0.23), (0.23–0.33), and (> 0.33) (Fig. 4m).

Soil texture is another effective factor used to specify sites suitable for recharge. Soil type is a major criterion in groundwater recharge and agricultural production. Soil texture provides essential information on infiltration rate^29^. For example, sandy soils have high permeability, which consequently might cause in reduced GWR. The soil map was obtained from Agricultural Research, Education, and Promotion Organization of Lorestan Province in 1:250,000-scale. The most prominent soil type in region were Loamy-sandy and Silty-loamy which cover 15.2% and 14.3%, respectively of the study area. The soil texture map of the study area was classified into 9 soil texture classes (Fig. 4n).

### Models

#### Maximum entropy (ME) model

We applied a family of machine learning techniques based on multinomial logistic regression know as maximum entropy (ME) to yield the potential of GWR from ground base observation and the condition factors. Jaynes introduced ME technique in 1957 based on probability distribution of maximum entropy to derive the probability of given number of individuals occurrence across the any given area based on a set of condition factors. The MaxEnt software (version 3.3.3 k), which is based on the ME approach, is conducted for GWR potential mapping. Groundwater potential mapping^63,64^, flood^30^, landslide^65,66^, rangeland^67,68^, earthquake^69^, species distribution^70^, springs and wells^71^, and groundwater quality^72^.

#### Frequency ratio (FR) model

The FR model, as the probability of incidence for a special attribute, is a simple geospatial assessment tool and is used to compute the eventual relation between GRW potential and GWR´s effective factors^30^. In specifying the frequency ratio, the recharge occurrence ratio in each conditioning factors sub-class is obtained toward the total recharge. Then, each class’s surface ratio is computed compared to the total area of the watershed.

The FR values were identified using Eq. (1) for each sub-class of GWR potential effective factors based on their correlation with GWR potential inventory:1
where α is the number of recharge pixels in each subclass, β is the total number of pixels in the area, γ is the total number of recharge pixels of the entire area. ɘ indicates the number of pixels in every subclass of conditioning factors, θ represents the percentage of recharge occurrences in any sub-class of conditioning factors, and ɷ is the relative percentage of the area of each subclass.

#### Ensemble modeling

In data analytics and predictive modeling, an individual model based on one data sample could have great variability, a large number of inaccuracies, or extensive biases, which affect the reliability of results^73^ (Rokach, 2010). The effects of these restrictions can be reduced by analyzing multiple samples or combining various models which can help provide better information to decision makers and improve modeling algorithms^39,73^. In the current study, we integrated the ME and FR models in various scenarios using basic mathematical operations (Table 1) in ArcGIS10.6.

**Table 1:** Predictive performance of models in ensemble scenarios.

Scenario	Ensemble model	AUC (0 to + 1)	CCI (0 to 100)
Scenario 1	(ME + FR)/2	0.987	83.1
Scenario 2	(2 × FR) + ME	0.987	76.7
Scenario 3	(2 × ME) + FR	0.984	54.6
Scenario 4	FR + ME	0.987	55.1
Scenario 5	FR × ME	0.990	90.7
Scenario 6	(ME + FR)/3	0.986	68.9
Scenario 7	((ME × AUC ME) + (FR × AUC FR)/(AUC ME + AUC FR))	0.984	83.8
Scenario 8	individual ME model	0.986	84.2
Scenario 9	individual FR model Odel	0.963	58.4

#### Conditioning factors importance and marginal response curves (MRCs)

The Jackknife test can be used to reduce bias of an estimator. This test removed one factor at a time and model created, here MaxEnt, with the remaining factors. In this study, the Jackknife tests were performed to determine individual conditioning factors importance for MaxEnt predictions. Accordingly, the influencing factor contribution in the analysis can be identified^74^. We also ran an MRC to quantify the conditioning factor’ behavior. MRCs were calculated and plotted for all 15 variables by the ME model to depict the role of each variable in the occurrence of GWRP in Marboreh Watershed. Plots represent the correlations of predicted sites with each independent variable relative to the dependent variable, showing how the individual predictor variables are related to the modeled class. On vertical axes of plots, values closer to 1 demonstrate the preferred range of the class.

#### Validation of the built models

The accuracy of predictive models should be analyzed by comparing the generated data with a training and testing dataset (existing GWR percolation points). To assess the performance of individual and ensembles models, we utilized the area under the Receiving Operator Curve (AUC-ROC)^74,75^ and the Correctly Classified Instances (CCI)^76,77^. The AUC value ranges from 0 to 1, in which the higher the value, the greater perfect discrimination^37^. The CCI derived from the corresponding confusion matrix that calculated true positive (TP), true negative (TN), false positive (FP), false negative (FN), with a range from 1 to 100. The greater CCI value, the more accurate prediction^78,79^.

## Result

### Conditioning factors importance

**Figure 5:**
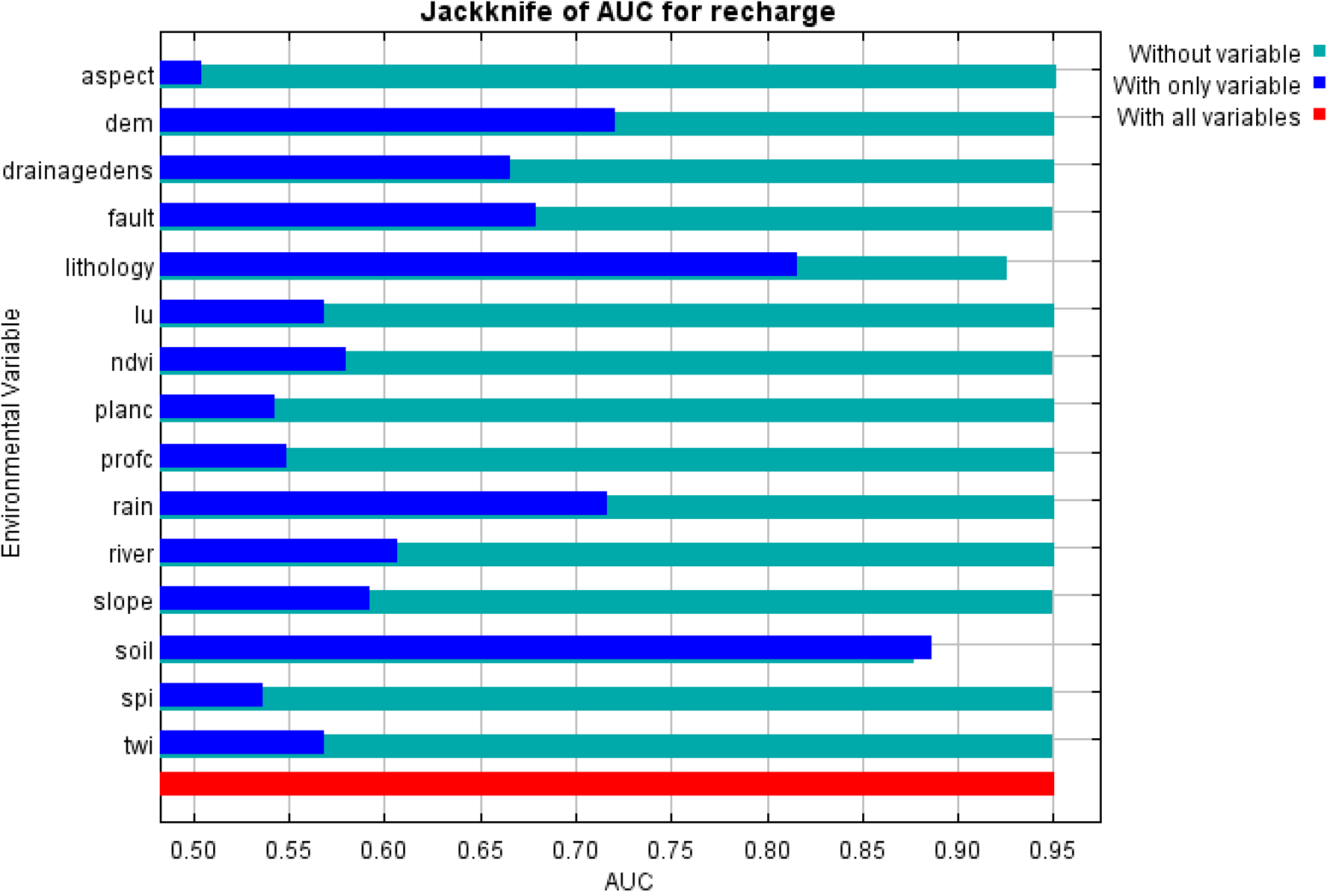
The Jackknife of regularized gain for GWR potential zones in Marboreh watershed.

The Jackknife test was implemented during MaxEnt model building to identify conditioning factors contribution (Fig. 5). According to Fig. 5, soil provided the most critical conditioning factor in GWR potential prediction, and lithology was second important having high training gain contained most unique information when they used independently. Also, DEM and rain conditioning factors provided high gain when used independently. While, distance from fault, drainage density, distance from river, slope and NDVI had moderate gains respectively, when used alone. Furthermore, soil and lithology reduced training gain the most when was excluded from the model, thus had the most information that were not present in other conditioning factors.

### Application of MaxEnt

**Figure 6:**
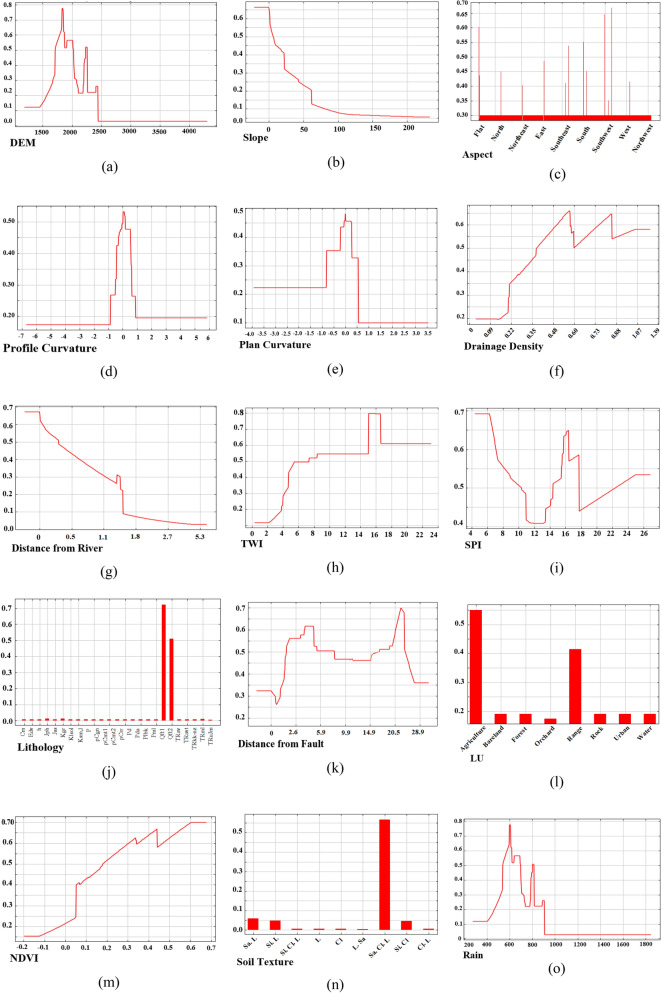
Marginal response curves for the quantitative conditioning factors (y-axis: predicted probability of GWR potential related to each conditioning factors). **(a)** Elevation; **(b**) slope degree; **(c)** slope aspect; **(d)** profile curvature, **(e)** plan curvature, **(f)** drainage density; **(g)** distance from river; **(h)** TWI; **(i)** SPI; **(j)** lithology; **(k)** distance from fault; **(l**) LU; **(m)** NDVI; **(n)** Soil texture and rain **(o)** (prepared in maxent 3.3.3 k software).

Firstly, we examined the response of GWR potential to effective factors. Response curves identified the quantitative relationship between the logistic probability of the GWR potential and effective factors. MRCs include curves representing the separate effect of each independent factor on the dependent variable (i.e. GWR potential). According to these graphs (Fig. 6), the highest probability of percolation was seen at elevations between 1810 and 1820 m, with a probability of 77% (Fig. 6a). These conditions are in accordance with the inverse relationship between elevation and percolation since percolation level is higher at the lowest points or in the plains, and decreases with increasing altitude. Due to the altitude range of the area, this elevation class has the most suitable conditions for GWR. Slope and aspect are also among the significant considerations in site selection for groundwater recharge. Steep slopes concentrate water on the lower slopes and contribute to the buildup of hydrostatic pressure^80^. Runoff on lower slopes has a better opportunity to concentrate and percolate. In Marboreh watershed, the slope layer was classified into five class and the highest probability (67%) was observed for the 1st class, which ranges from 0 to 4% (Fig. 6b). This slope range comprises the suitable slopes for recharging. Aspect does not directly affect runoff. Its role is in determining the rate of runoff generation due to the difference in microclimate on the different slopes. The highest probability of percolation (66%) was recorded for the southwest aspects (Fig. 6c). In order to determine the geomorphometric characteristics of shapes, the second derivative is used in digital elevation (curvature) model. This characteristic is associated with geomorphologic processes and has two distinct types of curvature with vertical properties that are called plan and profile curvature. Areas with concave morphology have the most potential for percolation. Concave morphology can concentrate water and moisture and create suitable areas for GWR^81^.

Permeability will be higher in areas with lower drainage densities because drainage by conduction of water on the ground prevents its penetration into the depths and thus reduces underground recharge^82^. The range for the drainage density factor at the studied watershed was between 0 and 1.39 km. The results showed that most places with recharge potential had drainage densities (with 67% probability) between 0.56 and 0.58 km (Fig. 6f). Results indicate that the probability of percolation in areas close to rivers was greater. The range of distance from rivers spanned 0–5.36 km (Fig. 6g). For the variable distance from river, the curve represents a steep decline with increasing distance, indicating that the more percolation area located near the river. In this study, the highest probability of percolation was observed at a distance of 0.52 km from rivers, with 0.67 percent. The curve shows a steep decline with increasing precipitation, indicating that this species likely prefers drier areas.

The TWI index is mainly used for quantitative topographic assessment of hydrological processes as well as showing the effect of topography on the position and size of groundwater flow, soil moisture, and saturated sources of runoff production. The higher index resulted in higher recharge potential (Fig. 6h). The stream power index, assuming the proportion of drainage to the surface area of the watershed, is a topographical combination feature. In this study, the highest percolation probability (69%) was seen in areas with SPI values in the 4–6 range (Fig. 6i).

The lithology of the studied area includes 26 geomorphic units. Areas with Qft2 quaternary lithology had the highest percolation potential because these layers are more closely associated with foothills and alluvial fans, and have high absorbance and percolation (Fig. 6j). This type of lithological unit, covering 33.65% of the watershed, is the largest lithological section in the region. Faults, due to the creation of crushed areas and the formation of water conduits, are very important in the development of morphology and the formation of subsidence in the region^54,83^. Faults facilitate the penetration of water to lower levels and increase the potential for the formation of underground liquidation cavities. The range of distance from faults was 0–28.9. The highest probability of percolation was 0.7% (Fig. 6k).

Croplands and rangelands (constituting 93% of the watershed’s land use) are known to be conducive to GWR due to presence of vegetation and permeable soil textures. According to the results, the highest percolation and recharge probability occurred in agricultural land and rangeland (Fig. 6l) where the soil crust of farmlands being broken by plowing and vegetation cover reducing the velocity of rain or surface water and providing more time for percolation. Cropland and rangelands, especially in areas with other suitable effective factors such as slope, elevation, and geology, show the highest recharge potential. Vegetation is a good sign of underground water and has a direct relationship with percolation potential; the greater the amount of vegetation in a region, the greater the permeability. NDVI was used to measure vegetation cover (ranging from 0.6 to 0.68). As depicted in Fig. 6m, the highest probability of recharge potential based on NDVI was found to be 70%. In Marboreh watershed, nine soil texture classes were identified. The sandy clay loam (Sa. Cl. L) class has the highest probability of percolation with 57% (Fig. 6n). Soil texture greatly influences water percolation, permeability, and water-holding capacity. The Sa. Cl. L class, due to its combination of coarse and fine grains, is one of the most permeable textures. According to the response curve of annual rainfall, the probability of GWRP occurrence (78%) increased in area with 600 mm and decreased smoothly after that (Fig. 6o).

### Application of FR model

**Figure 7:**
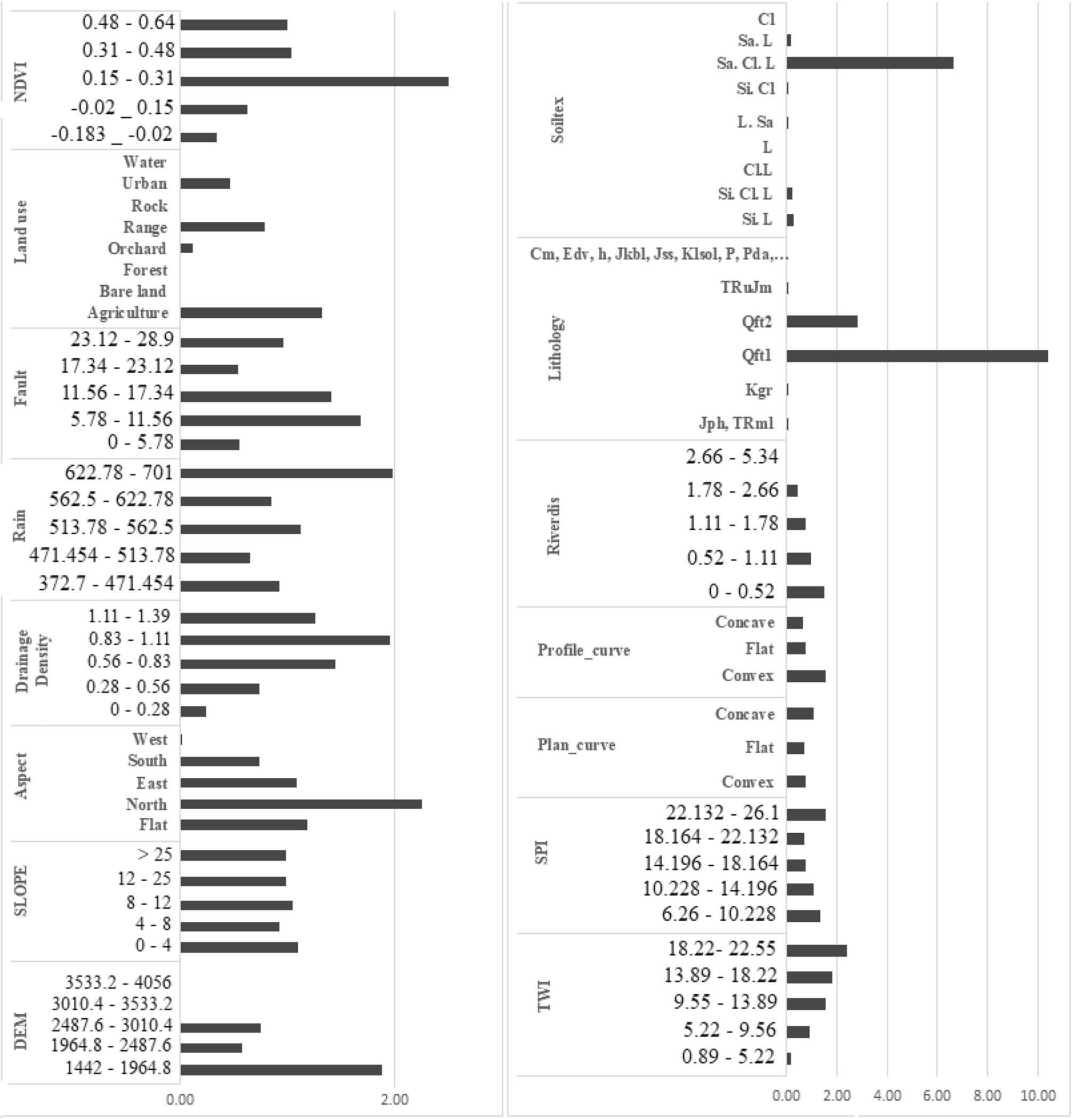
Spatial correlation between effective factors and GWR potential using FR model.

The outcomes of FR model calculated for each class of 15 effective factors based on their relationship with GWR potential locations are presented in Fig. 7, and a larger FR value means a higher probability on GWR potential of the corresponding effective factor. The ratio of the area with GWR potential to the entire area was calculated. In terms of elevation, high potential occurs mainly in the elevation range between 1442 and 1964.8 m. In the case of slope aspect, the FR weights were the highest for the north area (2.26) and flat area (1.19), while the West aspects have the lowest value of 0.01. For slope degree, the values of FR were decreasing through to increasing the slope degree. According to the FR, weights of profile curvature, convex, flat, and concave are 1.53, 0.77, and 0.64, respectively. For plan curvature, concave class had the highest GWR potential. In term of the drainage density, the class 0.28–0.56 had the greatest FR value of 1.96, while the class 1.11–1.39 had the lowest value of 0.24. Among various distance in the river network, the area closes to river had the most correlation with GWR potential, while the greatest distance from river had no GWR potential. TWI with higher values have larger weights of FR., while SPI with higher values have smaller weights of FR. The relationship between GWR potentials and the annual rainfall show that the high annual rainfalls have a high frequency of GWR potentials. The highest FR value (1.99) was observed for class > 622.5 mm.

Lithology FR conditioning factor was the highest in Qft1 (9.39) and has the highest FR value than other conditioning factors. Furthermore, Qft2 also had a high FR value of 2.82. In the study area, 0.40% and 33.7% of the area were covered by Qft1 and Qft2, respectively. Result showed that among the fault distance classes, the second class (5.78–11.56) and the third class (11.56–17.34) with FR values of 1.68 and 1.41 had the highest impact on GWR potential in Marboreh watershed. The results obtained in the present study showed that among the land use types, agricultural land (1.32) and range land (0.78) had the highest probability of GWR potential. According to the NDVI, the greater NDVI value had the higher potential for GWR. Another factor affecting GWR potential is the soil texture. The result revealed that the Sa. Cl. L soil texture weighing 4.66 had the highest probability on GWR potential and might indicate the high possibility of GWR potential in this thematic layer.

### Groundwater recharge mapping and validation of the built models

**Figure 8:**
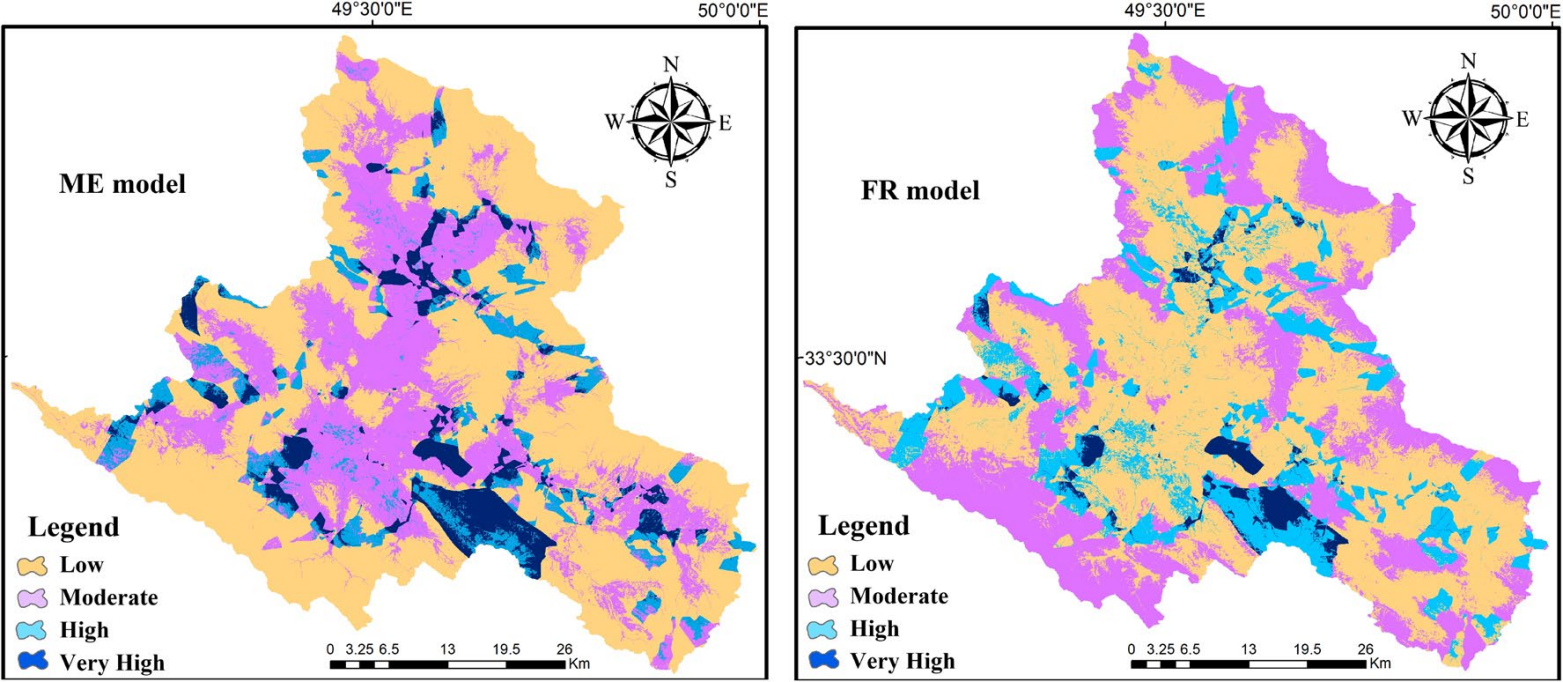
GWR potential zone map, **(a)** ME model and **(b)** FR model (maps layouts were prepared in ArcGIS 10.6 software).

The Maxent model performed well for predicting the GWR potential with the given set of training and test data with overall of 0.993 for AUC and 86.5 for CCI in testing data (Table 1). The GWR probability index was divided into four potential classes of low, moderate, high, and very high using a natural break method as seen in Fig. 8a. The model predicted approximately 7.26% of the studied region included very high GWR potential area (Table 2). The GWR with high and moderate potential encompass 8.59 and 29.45 percent of the studied area (Table 2). Through the AUC and CCI techniques the obtained map using the FR was evaluated. In the present study, the AUC value show 0.975 which revealed an “excellent” predictive accuracy in the model prediction. Also, the validation using CCI in total was 61.50% which has a satisfactory prediction. Ultimately, the map was generated and classified into four potential classes by natural break classification method, i.e., low, moderate, high and very high (Fig. 8b). Around 2.92% of the studied area falls into the very high GWR potential zone, while 36.63% of the land belongs to the low GWR potential category (Table 2). Based on the FR model, most parts of the studied area (45.20%) have a moderate GWR potential.

**Table 2:** The classes and area percentage of each class by scenario 5.

Class	ME	FR	Ensemble (Scenario 5)
Very high	3.7	0.4	3.01
High	5.29	11.7	8.84
Moderate	32.8	70.5	14.36
Low	58.21	17.4	73.79

### Application of hybrid model

The ensemble method, where a set of simple mathematic scenarios represented in Table 1 combining different base models to get better predictive performance compared to an individual model. Thus, to provide the GWR potential map with the ensemble model, the weights obtained from FR model and ME model were integrated to acquire a better GWR potential classifier. Seven ensemble classifier models were identified for GWR potential zones. AUC and CCI were considered as measures of models’ effectiveness. Table 1 showed AUC and CCI measures for seven classification models assessed. Compared with individual models (scenario 8 and scenario 9), it is relatively obvious that the proposed ensemble models outperformed with respect to AUC. CCI is also applied to assess the performance of the models, for which our results exhibited a satisfactory performance for GWR potential mapping. For the different scenarios, AUC and CCI ranged from 0.984 to 0.990 and 54.6 to 90.9, respectively (Table 1).

Effective classification of ensemble models in comparative to individual models indicated that the best ensemble classifier, scenario 5, is characterized by better validation measures in both training datasets and testing datasets. In addition, the results of both individual models clearly indicated satisfactory output for GWR prediction, while the FR model attained slightly lower predictive performance than ME model.

**Figure 9:**
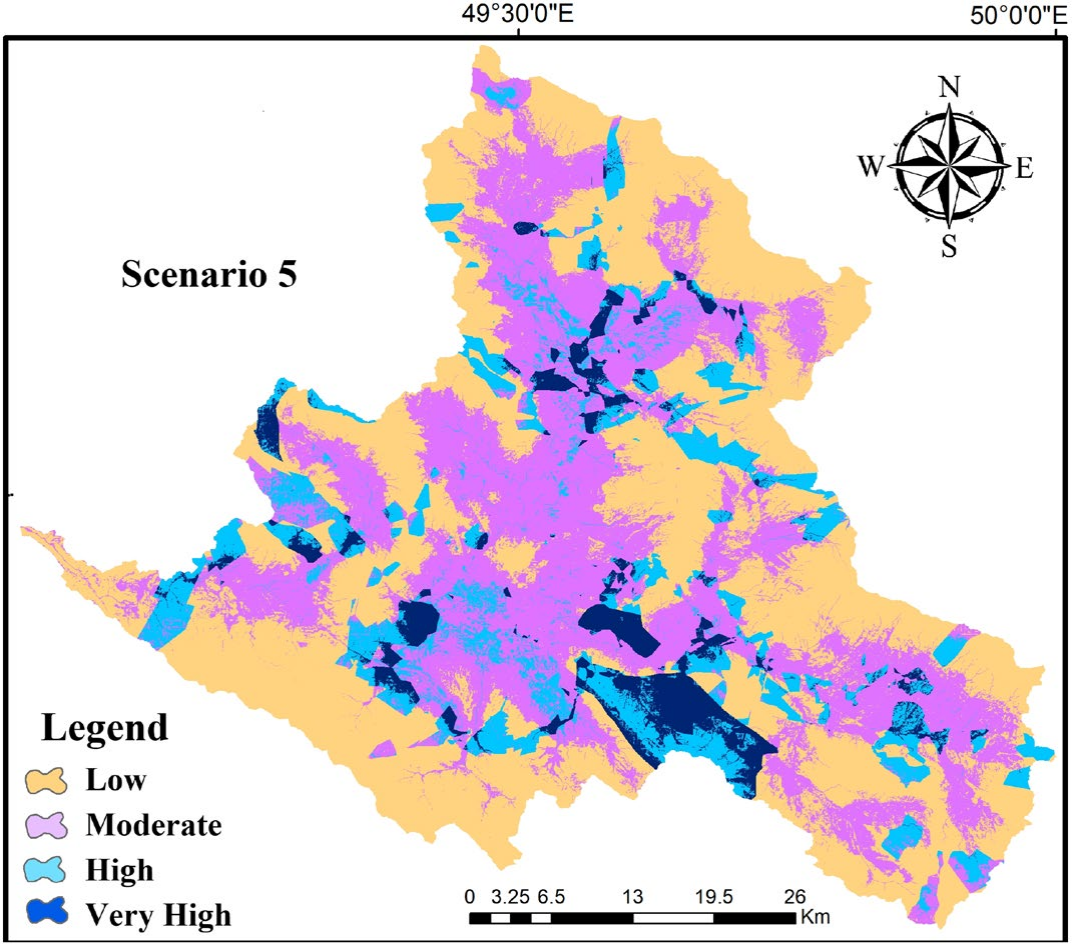
GWR potential zone map of ensemble method by scenario 5 (map layout was prepared in ArcGIS10.6 software).

The best ensemble model in terms of performance was scenario 5. Then, GWR potential map of scenario 5 was developed using ArcGIS10.6 software. Each pixel has a value that represents the occurrence of GWR potential. Consequently, the map was divided into four classes of low, moderate, high and very high based on natural break method (Fig. 9). The area of each class in scenario 5, was represented in Table 2. Based on the scenario 5, most parts of the studied area had a low GWR potential (73.79%). A very high GWR potential is only found in 3.01% studied area.

## Discussions

Nowadays, groundwater depletion became a critical global problem with serious consequences for sustainability of water supplies. Knowledge of spatial ground water recharge potential zones has placed considerable emphasis to addressing effectively planning and better managing water resources. Correspondingly, there has been growing numbers of research addressing zoning of groundwater recharge potential in recent years^19,27,29,36,37,54,55^. Although there still remains much work to understanding the effect of multiple drivers on the distribution and pattern of GWR potential. Population growth along with increased development led to heavy withdrawals from the plain aquifer system of the Lorestan province and subsequently decline in water table. Mountainous areas play a critical role in delivering water flow to the lowlands; therefore, it is extremely important to understand the GWR potential zones. Depending on hydrogeology, climate, physiography, and groundwater consumption, the volume of stored groundwater may vary considerably in different places^19,29,84,85^. While physical models are promising to quantitative estimation of natural recharge rates to aquifer system, they often require large hydrogeological data which are not easily measurable in the field^86^ and require numerically intensive simulation of the behavior. Furthermore, in-depth physical knowledge of the relevant hydrological process is requiring when developing physical based models (Kim et al., 2015). Moreover, many developing countries suffer from lack of information, accordingly susceptibility and potential mapping with help of statistical methods and machine learning methods which requiring few parameters without an explicit need about the underlying physical process becomes a useful tool to subdivide area into regions with significant GWR potential zone. Nevertheless, significant improvements in model prediction and decreased the prediction uncertainty through an ensemble approaches were recently published by researchers^32,41,44,46^. The current state of scientific researches on disaster susceptibility and natural potential by an ensemble models are in the early stage of development.

In our study, GWR potential zones were investigated in Marboreh watershed in Lorestan province, Iran, using a combination of the machine learning (ME) and a bivariate statistical method (FR), into one predictive model in order to improve predictions. These two individual methods are commonly used by researchers in the field of Groundwater potential mapping^41,43^, landslide^47,48^, flood^44,45^. In order to make clearly accurate evaluations of the ensemble methods, FR and ME were also performed independently. The result showed that the ensemble method in scenario 5 performed “excellent’ with AUC and CCI values of 0.990 and 0.907, respectively. This supports the previous findings from Tehrany et al^74^; Razavi-Termeh et al^28^ and Di Napoli et al^87^ that ensemble classifiers allow more accurate prediction than classical models and alternative to the individual models in susceptibility and potential mapping. A research carried out in Qued Guenniche in the north of Tunisia to produce a GWR potential map by Chenini and Msaddek^36^. The authors found that the bivariate and multivariate statistical approaches provide more accurate results than the AHP. Pourghasemi et al^37^compared a number of machine learning methods to investigate groundwater recharge potential zones in Firuzkuh County, Iran. The results showed that SVM and MARS outperformed RF in terms of accuracy based on the data from 2000 double ring infiltrometer.

Here, we also address importance effective factors of GWR potential. Precisely, to quantify effects of topographical, hydrological, geological and ecological factors on GWR potential using data from 850 observations. Jackknife test was applied to evaluate the relative importance of each causal factors. The result indicated that soil had the highest training gain. This is followed by lithology, aspect and DEM. Our findings are in agree with pervious researches that highlight the influence of the soil, lithology, elevation, aspect and drainage density in GWR potential identification^29,55^. Similar results were found by Pourghasemi et al^37^ in the Firuzkuh County, Iran, who noted that the mean annual rainfall, drainage density, elevation and slope angel are dominate factors that influence groundwater recharge. Rainfall data have been widely used in understanding the GWR potential zones^21,25,29,37,55,88^. The spatial distribution of the final GWR potential map is consistent with the findings of several studies. Patil and Mohite^88^, Senanayake et al^29^, Yeh et al^25^, de Costa et al^21^ and Pourghasemi et al^37^ obtained the high effective GWR potential in gentle slope. In contrast, the least GWR potential can be found in higher drainage density zones^25,54^. Additionally, high altitude along with steep slope areas could identified poor GWR potential. Furthermore, based on Jackknife test, SPI, plan and profile curvatures have no particular impact on GWR potential in studied area. Moreover, the final GWR potential maps indicated that the areas with high percolation potential are mainly distributed along the south and southwestern parts of the study area.

## Conclusion

Marboreh Watershed continue to an experience water scarcity, largely driven by frequent droughts and expanding agricultural land combined with over abstraction of groundwater. Therefore, there is a need to identify potential groundwater recharge zones to help prevent water scarcity. In this study to address water scarcity issue we discuss the combination of the predictions achieved by FR and MA models in the ensemble in order to develop a GWR potential zones for the Marboreh watershed in Lorestan province, Iran. Moreover, the spatial mapping for managed aquifer recharge based on topographic factors, hydrological factors, geological factors and ecological factors and assessing their likely contribution on GWR is explored. The results of the research reveal that evaluating the individual’s models using the AUC and CCI indices reach relatively high performance. The ensemble classifier with scenario 5 may achieves more accurate result than other ensemble models and individual models with AUC and CCI values of 0.990 and 0.907, respectively. Additionally, GWR potential areas were found to vary largely based on soil, lithology, aspect and elevation. The results of the study revealed that about 11.85% of the Marboreh watershed was found to have a very high to high recharge potential and 73.79% falls in the low zone class.

It has been generally recognized that, the accuracy of the outputs of interest is dependent mainly on the input uncertainty. There are large uncertainties in soil spatial information, insufficient knowledge of underlying groundwater system and inadequate of data measurements. Additionally, higher uncertainties are associated with aquifer characteristics and recharge process needs to be address of further studies. Here, since there were no available data for hydraulic conductivity, double-ring infiltrometer method was used to identify varying groundwater recharge potential. Thus, priority needs for validate model predictions in area with monitoring wells and resistivity imaging of water movement in vadose zone.

The results of this study could be used in the planning, management, and control of surface runoff in high-discharge events. It is recommended to construct aquifer recharge facilities in areas with “high” and “very high” percolation potential. Using these findings, it is possible to obtain higher quantities of surface water and recharge aquifers, as well as managing water deficit. The studied area heavily relies on the abstraction of groundwater to sustain irrigated agriculture. Consequently, identifying the groundwater recharge zones is promising, especially for future droughts, but requires more scientific support and supervision to succeed. The studied area is suffering from a combination of water crisis and groundwater pollution. Therefore, identifying zone with a high GWR potential is critical to enhance water storage and taking acting by local managers to define conservation priorities areas to reduce nutrient losses and enhances aquifer recharge.
